# Gunshot Wound of Uterus; Bullet Traversing Six Months’ Fœtus; Recovery of Patient in Four Weeks

**Published:** 1879-10

**Authors:** Geo. B. Hays


### GUNSHOT WOUND OF UTERUS; BULLET TRAVERSING SIX MONTHS' FŒTUS; RECOVERY OF PATIENT IN FOUR WEEKS.

By Geo. B. Hays, M.D.

The late Professor Paul F. Eve, in his work entitled "Remarkable Cases in Surgery," on page 581, under the heading "Remarkable Wounds and Injuries," quotes from the *Boston Medical and Surgical Journal*, 1853, a case reported by Dr. Palmer, of the East India Company's service, in which a Burmese woman received a "bullet wound of the bladder and womb," and "recovered in three weeks." As Dr. Palmer's patient received the wound in an unimpregnated uterus, and the case was cited as an instance of great recuperative power and recovery from wounds ordinarily fatal, I would like to place on record a very similar case which recently came under my observation, in which the complications were far more grave, and which also resulted in a complete recovery.

June 20th, 1879. Was summoned hurriedly to Magnolia Plantation, six miles distant, to see Mary Washington, colored, aged 18, married, six months pregnant, primipara, who was reported as suffering from a gunshot wound of abdomen received that morning. Reached the place at 9 A.M. and learned that about three hours previous the assistant overseer had fired a pistol shot at some hogs in the cane field; the ball had *ricochetted* from the hard, dry field road, and wounded the woman, who was standing concealed behind a clump of tall alder bushes sixty yards distant from where the shot was fired. Upon examination I found the patient beginning to react from the shock, and complaining of severe pains in the abdomen.

The ball (one from the same cartridge box weighed 136 grain) had penetrated the abdominal cavity at the left side, about two inches diagnoally in front and above the anterior superior spinous process of the ileum, ranging upwards—confirming the statement that it had first struck the ground, shot having been fired from on horseback, and had lodged within the abdominal cavity.

There had been but very little hæmorrhage externally at the first, and I found a portion of omentum an inch in length protruding from the wound; completely plugging it. The woman had not menstruated since December, and stated she was six months advanced in pregnacy. It was evident the abdominal wall was cut through, and furthermore, if the projectile had sufficient velocity at the moment striking the uterus and contents would be involved in the injury, but the distance from which the shot was fired, and the fact of the ball first striking the ground, prevented a positive diagnosis at the moment, probing of course being inadmissible. I reduced the protruded omentum and turned the woman upon her left side, in order to allow drainage in the event of internal hæmorrhage or effused liquor amunii in the abdominal cavity. Prescribed full doses of sulph. morphia, and ordered large, warm linseed poultices, abundantly saturated with laudanum, to be constantly applied to the abdomen.

June 21st. Visited patient at 10 A.M. She had well marked labor pains which had begun to come on about sunrise, as stated by the nurse. She had rested badly all night, scarcely slept at all. Entire abdomen extremely tender and distended. Could not bear light percussion. During a pain would by an effort of will control and arrest the contraction of the abdominal muscles, thereby throwing all the work upon the uterus. Shortly after 11 A.M., the contractions being stimulated by fluid extract ergot, the fœtus, placenta and membranes were expelled simultuneously with very slight gush of waters when the membranes were ruptured. Some coagula escaped with the fœtus. After delivery the uterus contracted beautifully, not more than two ounces of blood being lost. Administered alcoholic stimulants and beef-tea, the patient being much exhausted. Continued the poultices and fastened the bandage over them. Examined the fœtus for the pistol ball and found it had penetrated beneath the left scapula, ranged diagonally through the trunk a distance of about three inches, and made its exit in the right hip. Careful search could not find the ball either in the placenta or among the coagula, and I was forced to the conclusion that it had entirely traversed the uterus, and as it could not be felt externally on the right side that its course had been arrested just as it attained the inner surface of the abdominal parietes on that side. The child was a female, ten inches in length, well developed, nails formed, eyelids adherent. Evidently a six or six and a half months fœtus.

Puerperal fever set in, accompanied by peritonitis. Opium, quinine and calomel were the medical agents principally relied upon, and the poultices before mentioned were continued until recovery. Laxative enemas were sometimes resorted to. For the first few days of her illness I had no expectation of her recovery, and in fact more than once carried with me on my visit the necessary instruments to make an autopsy, expecting to find her moribund. In my treatment I was actuated greatly by the motto *Dum anima est, spes est,* and placed a deal of reliance upon the *vis medicatrix natarœ.*

From the 27th of June, her general condition underwent a change for the better and she steadily continued to improve. The opium, quinine and calomel were persisted in until her gums became touched, when the latter was discontinued, and a solution of chlorate of potash used as a mouth-wash. A strong camphor ointment relieved the inflamed breasts. She had very little lochial discharge, it lasting only two or three days. There was no drainage from the external wound at all, and it was closed with a strip of plaster and subsequently, when it suppurated a little, dressed with oxide zinc ointment.

July 17th, she began menstruating with but little pain or discomfort; she ceased on the 19th, and the following day, July 20th, just one month from the date of the injury, she was dismissed, well. It is proper to state here that the manager of the plantation kept an experienced nurse at the bedside day and night, and kept the patient supplied with everything necessary for her welfare, which undoubtedly contributed very greatly toward her recovery. She is now, August 9th, walking about, feels well, sleeps well, has the appetite of a tramp, and suffers no inconvenience from the presence of the ball in her internal economy.

It would be interesting to know exactly by what physiologcal process the liquor amnii that escaped into the cavity of the abdomen became so readily absorbed, while the entire cavity was in such an altered and abnormal condition.—*New Orleans Medical and Surgical Journal.*

